# Tightly Regulated Expression of *Autographa californica* Multicapsid Nucleopolyhedrovirus Immediate Early Genes Emerges from Their Interactions and Possible Collective Behaviors

**DOI:** 10.1371/journal.pone.0119580

**Published:** 2015-03-27

**Authors:** Chikako Ono, Masanao Sato, Hitomi Taka, Shin-ichiro Asano, Yoshiharu Matsuura, Hisanori Bando

**Affiliations:** 1 Laboratory of Applied Molecular Entomology, Division of Applied Bioscience, Graduate School of Agriculture, Hokkaido University, Sapporo, Japan; 2 National Institute for Basic Biology, Okazaki Institute for Integrative Bioscience, National Institutes of Natural Sciences, Higashiyama, Myodaiji, Okazaki, Japan; 3 Department of Molecular Virology, Research Institute for Microbial Diseases, Osaka University, Osaka, Japan; Ecole des Mines d'Alès, FRANCE

## Abstract

To infect their hosts, DNA viruses must successfully initiate the expression of viral genes that control subsequent viral gene expression and manipulate the host environment. Viral genes that are immediately expressed upon infection play critical roles in the early infection process. In this study, we investigated the expression and regulation of five canonical regulatory immediate-early (IE) genes of *Autographa californica* multicapsid nucleopolyhedrovirus: *ie0*, *ie1*, *ie2*, *me53*, and *pe38*. A systematic transient gene-expression analysis revealed that these IE genes are generally transactivators, suggesting the existence of a highly interactive regulatory network. A genetic analysis using gene knockout viruses demonstrated that the expression of these IE genes was tolerant to the single deletions of activator IE genes in the early stage of infection. A network graph analysis on the regulatory relationships observed in the transient expression analysis suggested that the robustness of IE gene expression is due to the organization of the IE gene regulatory network and how each IE gene is activated. However, some regulatory relationships detected by the genetic analysis were contradictory to those observed in the transient expression analysis, especially for IE0-mediated regulation. Statistical modeling, combined with genetic analysis using knockout alleles for *ie0* and *ie1*, showed that the repressor function of *ie0* was due to the interaction between *ie0* and *ie1*, not *ie0* itself. Taken together, these systematic approaches provided insight into the topology and nature of the IE gene regulatory network.

## Introduction

The initiation of signal transduction is one of the most important steps during any biological process, because it may determine how subsequent signaling events behave and the outcomes of the signaling process. In particular, successful initiation of signal transduction is required for biological systems to appropriately respond to external stimuli or perturbations. Likewise, pathogens need effective strategies and components for their own signaling programs to invade hosts that have anti-pathogen responses. Robustness, the property to generate reproducible outputs under various conditions despite perturbations, appears to be particularly important for the successful initiation of the signaling process.

Baculoviruses, double-stranded DNA viruses with large genomes, have relatively narrow host ranges, but are highly adapted to hijacking the host cellular machineries. The extraordinarily high expression of baculoviral proteins late in the infection process has been exploited for recombinant protein expression [1]. The expression of baculoviral genes is sequentially regulated, and these genes are classified based on their expression timing during infection: immediate early (IE), delayed early, late, and very late. Some viral genes expressed in the immediate early phase encode proteins involved in the transcriptional regulation of viral genes expressed in later phases [2–4]. The genome of the most studied baculovirus, *Autographa californica* multicapsid nucleopolyhedrovirus (AcMNPV), encodes IE genes such as *ie0*, *ie1*, *ie2*, *gp64*, *he65*, *me53*, *p35*, and *pe38*. Five IE genes *ie0*, *ie1*, *ie2*, *me53*, and *pe38* have validated or inferred transcriptional regulatory functions and have been studied as canonical regulatory IE genes [5–9]. Among these, the regulatory functions of *ie1*, *ie0*, *ie2*, and *pe38* have been studied previously. IE1 is an acidic transcriptional activator that regulates viral gene expression globally [1,10–12] and is essential for viral DNA replication or viral proliferation [2–4,13]. IE0 is one of the late expression factor genes [14], and its primary structure is identical to IE1, except for 54 amino acids at the N-terminus that are added as a result of transcriptional splicing. The IE0 of another baculovirus, OpMNPV, activates the promoters of early genes, such as *ie1* and *gp64* [15]. IE0 in LdMNPV also activates transient transcription and DNA replication [16]. IE2 may be involved in the transcriptional activation of *ie1*, *38k*, and its own promoter [17]. In addition, IE2 is predicted to have a RING finger structure that is involved in E3 ubiquitin ligase activity and in interactions with E2 ubiquitin-conjugating enzymes [18]. It is also involved in cell cycle regulation [19]. PE38 also contains a RING finger motif, which is essential for E3 ubiquitin ligase activity [18]. PE38 may be involved in DNA replication [13] and/or transcriptional activation [9,20]. OpMNPV PE38 also has transactivation activity on a viral gene promoter [21]. In contrast to the other four IE genes, the transcriptional regulatory function of *me53* has yet to be determined; however, ME53 has a predicted zinc finger motif, which suggests a DNA sequence–specific binding function [8]. In addition to these IE genes, *gp64* and *p35* are also expressed during the immediate early stage [3,22]. gp64 is a structural protein [23–26] and does not have a reported transcriptional regulatory function, whereas p35 enhances late gene expression [27]. However, p35 does not contain any known domains with a transcriptional regulatory function, and the enhancement of late gene expression is mediated by stimulating replication of the DNA template for viral gene transcription [28].

IE genes regulate not only the expression of late genes [4,11], but also that of other IE genes [12,29]. Transient expression analyses, in which the regulatory activity of a gene on a promoter is analyzed in isolation, have been used to examine the regulatory relationships among IE genes. IE1 negatively regulates the transcription of *pe38* and *ie2* [29], whereas it stimulates its own promoter and represses expression at the *ie0* promoter. IE0 transactivates the *ie1* promoter, but does not affect expression from its own promoter [12]. Genetic approaches using bacmids, cloned baculoviral genomes, have provided information on the functions of IE genes during infection. A recent study using the AcMNPV mutant for *pe38* revealed the delayed expression of *ie1*, which demonstrated that PE38 directly or indirectly regulated *ie1* transcription [30]. A recombinant AcMNPV with a mutated *ie0* promoter reduced the expression of *ie0* and increased steady-state levels of IE1 to higher than those of IE0 [20]. These findings indicate that the regulatory relationships between IE genes predicted by transient expression analyses may not necessarily correspond to those found during viral infections.

Our current knowledge of the regulatory relationships among AcMNPV IE genes is largely based on observations made in separate studies performed in different laboratories and/or in different cell lines. Caution must be exercised in interpreting these findings because of the possible context-dependent behaviors of IE gene regulation in each experiment. The contribution of IE0 to successful infections differs in a cell line–specific manner [31]. Even when cell lines with the same origin are used, long-term culture in different laboratories changes the properties of cell lines and affects baculoviral infections [32]. Therefore, a systematic analysis of IE gene regulation under controlled conditions is required to avoid possible contextual differences, as well as to provide more appropriate information for building a model of the IE gene network.

Here, we describe how the expression of the five canonical regulatory IE genes, *ie0*, *ie1*, *ie2*, *me53*, and *pe38*, is regulated by the AcMNPV IE gene regulatory network. We modeled the topology of the IE gene network using systematic transient expression and genetic analyses on the IE genes. The results revealed two prominent features of IE gene expression and regulation: (1) IE genes are generally transactivators, and (2) IE gene expression in early infection is tolerant to single deletions of activating IE genes. This modeled network topology suggests that the robust control of IE gene expression could be partially attributed to how each gene is regulated by other IE genes and host genes. In addition, our analyses suggest that interactions between IE genes may play important regulatory roles in the IE gene expression. Combinatorial genetics and statistical modeling [33,34] on *ie0* and *ie1* revealed that the IE0–IE1 interaction repressed other IE genes, perhaps to maintain steady-state expression levels. Based on the significant contribution of IE gene interactions to viral gene expression, a combinatorial reconstitution of the network [33,35] to identify interactions among viral genes will be necessary to advance our understanding of viral gene function and molecular mechanisms during infection.

## Materials and Methods

### Cells, virus, and transfections

The infectious AcMNPV bacmid bMON14272 (Invitrogen) was propagated in *E*. *coli* strain DH10B, as described previously [36]. Sf9 cells were maintained in TC-100 medium (Sigma) containing 10% FBS at 26°C. Transfection of Sf9 cells with viral or plasmid DNA was performed by lipofection using the FuGENE HD Transfection Reagent (Promega), according to the manufacturer’s instructions. In order to control for batch effects in the plasmids or the bacmids, we extracted them at one time and stocked them as aliquots for each experiment.

### Construction of plasmids for transient expression analysis

#### Construction of reporter plasmids

**Fig 1:**
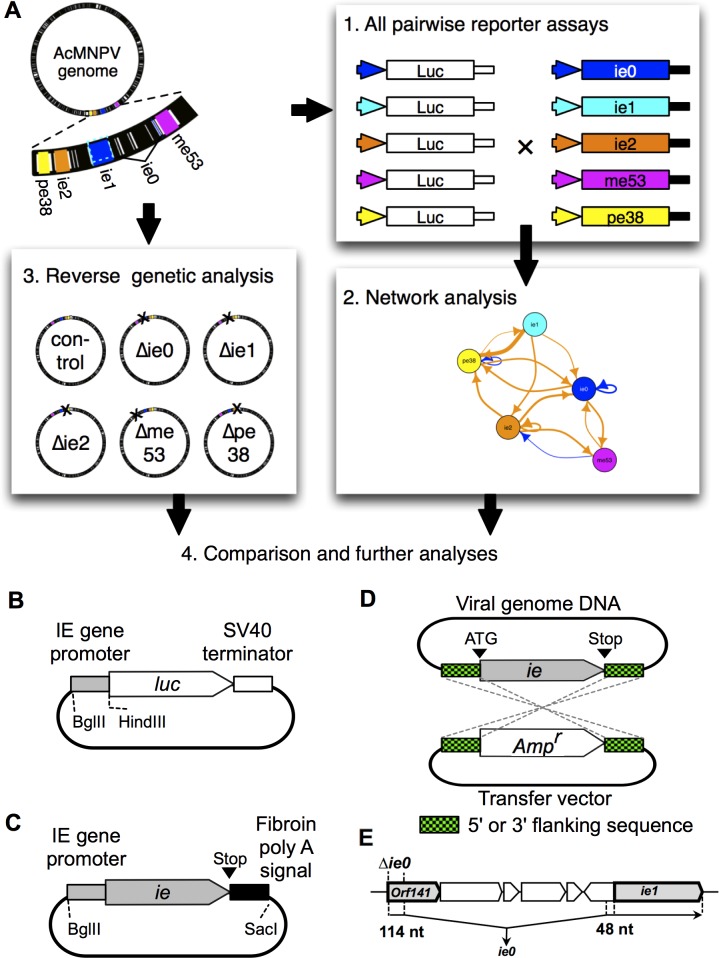
Study and construct designs. (A) Study design. *ie0*, *ie1*, *ie2*, *me53*, and *pe38* were selected because they are validated or inferred regulators of IE gene expression. 1, Plasmids for transient expression analysis were constructed from the AcMNPV-C6 genome. Transient expression analyses to measure the IE gene promoter activities were performed in all combinations of the five IE gene expression cassettes and six reporter plasmids (five containing IE gene promoters and one empty vector). The results obtained from this were used in a network analysis (2). 3, Reverse genetic analyses were performed using bacmids that each lacked one of the 5 IE genes. IE gene transcription in the knockout viruses was quantified using quantitative real-time PCR. 4, The results obtained in the transient expression analysis and the genetic analysis were compared and used to build and test hypotheses. (B and C) The designs of constructs used for transient expression analysis. (B) The reporter plasmids. Approximately 500 bp of the sequence upstream of the IE ORFs were used as the IE gene promoters in this study. (C) IE gene expression plasmids. The IE gene ORFs and the upstream sequences used in the reporter plasmids were inserted into a pGEM-Teasy vector carrying a fibroin poly(A) signal. (D) Schematic representation of the generation of IE gene knockout viruses. The entire IE gene ORFs, from the initiation codons to the termination codons, were replaced with an ampicillin resistance gene. (E) The structure of immediate early ie0 transcript and the genomic region used to knock out *ie0*. Immediate early ie0 transcript consists of two exons. Δ*ie0* bacmid was generated by replacing a part of *Orf141* ORF with an ampicillin resistance gene.

To construct reporter plasmids that express luciferase under the control of each IE gene promoter, approximately 500 bp of upstream sequence was amplified by PCR with the primer sets in S1 Table. The IE gene promoter sequences were ligated into the plasmid vector pGEM-Teasy (Promega) to produce intermediate vectors. The IE gene promoter sequences were excised from the intermediate vectors by digestion with BglII and HindIII, and were then ligated to the BglII-HindIII site of PGV-p (Toyo Ink MFG. Co. Ltd.) to obtain PGV-ie0p, -ie1p, -ie2p, -me53p, and -pe38p (Fig. 1B).

#### Construction of IE expression plasmids

Plasmids that express each IE gene under the control of its native promoter were constructed. For *ie1*, *ie2*, *me53*, and *pe38*, each ORF and ~500 bp of its upstream sequence were obtained from the AcMNPV C6 strain by PCR using the primer sets listed in S1 Table. Each of the amplified sequences, and a fibroin polyadenylation signal flanked by PstI and SacI cut sites, were ligated into the BglII–SacI site in pGEM-Teasy (Fig. 1C). These plasmids were designated pGEM-*ie1*, -*ie2*, -*me53*, and -*pe38*. We cloned an *ie0* isoform that was expressed during the immediate early phase. Immediate early–phase *ie0*, generated by splicing, consists of the partial 5´ end of exon0/orf141, a partial 5´ UTR, and a complete ORF of *ie1*. In order to express immediate early–phase *ie0* from the sequence coding both *ie0* and *ie1*, we introduced a mutation into *ie0* at the codon of the 55th amino acid of IE0 (ie0M55A), which corresponds to the initiation codon of IE1. This mutation results in the suppression of translation beginning at that sequence. Mutagenesis was performed by PCR using the primers Acie0 5' Hind, Acie1 3' Pst, and Acie0-M55A. The *ie0* promoter region from pGEM-05 and the mutated *ie0* were inserted downstream of the *polh* ORF of pFastBac-AcPolh (see the section of “Rescue of the polyhedrin gene [*polh*] with and without mutations to suppress *ie1* [Δie1] in bacmids.”) to generate pFastBac-AcPolh-AcIE0full-M55A. The *ie0* sequence, including the ORF and 500 bp of upstream sequence, was excised with BglII or PstI from pFastBac-AcPolh-AcIE0full-M55A and ligated into pGEM-Teasy, as described above, to obtain pGEM-*ie0*.

### Transient expression analysis

Sf9 cells at a density of 4 × 10^5^ were co-transfected with 0.5 μg of a reporter plasmid (PGV-ie0p, -ie1p, -ie2p, -me53p, or -pe38p) and 0.5 μg of an IE gene expression plasmid (pGEM-*ie1*, -*ie2*, -*ie0*, -*me53*, and -*pe38)*. Cells were cultured in TC-100 medium for 48 h. A self-ligated pGEM-Teasy vector (pGEM) was used as a control plasmid. The quantification of luciferase activity in the transfected cells was performed as previously described [37]. Each measured value was obtained by subtracting the background signal from the measured signal. A set of experiments consisted of 60 measurements (30 for each pairwise combination of the six luciferase plasmids and five IE gene expression plasmids, and two independent biological replicates). Two independent sets were performed. Log_10_-transformed measured values were fitted to the mixed linear model using the nlme package (version 3.1–117) in R (version 3.0.3):

$$V_{ijkl}\;=\;P_{i}+{P:I}_{ij}+{S:R}_{kl}+\varepsilon_{ijkl}$$

where *V*, *P*, *I*, *S*, *R*, and *ε* correspond to the log_10_-transformed measured value, the promoter, IE gene, set, replicate, and residual, respectively. *P* and *I* are fixed factors. *S*, *R*, and *ε* are random factors. The sum of the mean estimates of the promoter effect and the promoter:IE gene interaction was used as the modeled measured value. The *p*-value associated with the estimated promoter:IE gene interaction was corrected using the Benjamini–Hochberg False Discovery Rate [38] to obtain the *q*-value. The *q*-value was used to evaluate if the promoter:IE gene interaction was statistically significant (*α* = 0.1).

### Graph analyses

Shortest path length and normalized betweenness were calculated using R (version 3.0.3) and the igraph package (version 0.7.1). A custom R script was used to simulate single-gene perturbations.

### Construction of IE gene–targeting plasmids

The AcMNPV bacmid system and homologous recombination [36,39] were used to generate IE gene–knockout viruses. We generated transfer plasmids containing each IE gene locus sequence, with the ORF replaced by an ampicillin resistance gene. Briefly, the 5´ or 3´ non-coding regions (0.5–0.8 kbp) of the AcMNPV genes were amplified using PCR with the primer sets in S1 Table. The sequences 5’ and 3’ adjacent to the *ie0*, *ie1*, *ie2*, *me53*, or *pe38* ORFs were ligated into the plasmid vector pGEM-Teasy (Promega) to produce pGEM-05, -15, -25, -Me5, and -Pe5 or pGEM-03, -13, -23, -Me3, and -Pe3, respectively. An ampicillin resistance gene was excised from pGEM-3Zf(-)-Ampr (Ono, unpublished) using SalI and HindIII. The 5’ non-coding sequences of *ie0*, *ie1*, *ie2*, *me53*, and *pe38* were excised by digestion with HindIII and BglII from pGEM-05, -15, -25, -Me5, and -Pe5. The DNA fragments containing the 3´ non-coding sequences of *ie0*, *ie1*, *ie2*, *me53*, and *pe38* and an ampicillin resistance gene were ligated into pGEM-05, -15, -25, -Me5, and -Pe5 using the HindIII, SalI, and SpeI sites, which resulted in pGEM-Δie0, -Δie1, -Δie2, -Δme53, and -Δpe38, respectively.

### Construction of IE gene KO bacmids

To generate IE gene knockouts (KOs), we used AcMNPV bacmids and homologous recombination (S1B Fig.) in Sf9 cells. Briefly, Sf9 cells seeded at a density of 1×10^6^ in a 6-well plate (Iwaki) were co-transfected with 1 μg of bacmid DNA and 3 μg of the targeting plasmid pGEM-Δie0, -Δie1 (to knock out *ie0* and *ie1* simultaneously, called the *ie0ie1*-knockout), -Δie2, -Δme53, or -Δpe38. After being incubated for 5 days, the recombinant bacmid DNA was purified from the transfected cells. Twenty μl of ElectroMAX DH10B cells (Invitrogen) was transformed with the recovered bacmid by electroporation at 25μF, 2.5kV, and 200Ω using a GenePulser (Bio-Rad). Each IE gene KO AcMNPV bacmid (S1 Fig.) was obtained from ampicillin-resistant, kanamycin-resistant, and lacZ-positive colonies, followed by verification of the absence of the IE gene ORF by PCR (S2 Fig.). The primer sets used in the verification targeted an interior 300-nucleotide region in each IE gene ORF (S1 Table).

### Rescue of the polyhedrin gene (*polh*) with and without mutations to suppress *ie1* (Δie1) in bacmids

To generate *polh*-rescued, IE gene–knockout bacmids, *polh* and its promoter were obtained from the AcMNPV C6 strain by PCR using the primers polh-F and polh-R. The PCR product was cloned into pGEM-Teasy (Promega) and was excised using EcoRI and SpeI before subcloning into the transfer vector pFastBac1 (Invitrogen). The resultant plasmid was designated pFastBac-AcPolh.

DH10Bac cells containing the helper plasmid pMON7124 were transfected with each bacmid by electroporation as described in Ono *et al*. [39]. The transposition of *polh* or *polh*-*ie0*M55A to each IE gene–knockout bacmid in the bacterial cells was performed with pFastBac-AcPolh (for *ie0*-knockout, *ie0ie1*-knockout, *ie2*-knockout, *me53*-knockout, and *pe38*-knockout bacmids) or pFastBac-AcPolh-AcIE0full-M55A (for the *ie1* knockout bacmid; S1B Fig., see the section “Construction of IE expression plasmids”). The bacmid DNA carrying *polh* or *polh*-*ie0*M55A was isolated from a kanamycin-resistant, gentamycin-resistant, and lacZ-negative colony, and designated AcBac+polh (AcP+), AcBac+polh-ie0KO (Δie0), AcBac+polh-ie1KO (Δie1), AcBac+polh-ie0ie1KO (Δie0/ie1), AcBac+polh-ie2KO (Δie2), AcBac+polh-me53KO (Δme53), and AcBac+polh-pe38KO (Δpe38), respectively. The rescue of *polh* or *polh*-*ie0*M55A in IE gene KO bacmids was verified by PCR using the primer pairs ORF008-5 and ORF008-300, or ORF141-5 and ORF147-300 for targeting *polh* and *ie0*, respectively (S1 Table).

### Quantitative real-time PCR analysis

The expression level of each IE gene was measured with quantitative real-time PCR. Sf9 cells were seeded on 6-well plates at a density of 1×10^6^ per well and were transfected with 2 μg of each IE gene–knockout bacmid DNA. Total RNA was isolated from the transfected Sf9 cells 6 hours post-transfection (hpt) using the TRIzol reagent (Invitrogen). The total RNA was treated with DNase I and RNase inhibitors (TaKaRa), and was purified by ethanol precipitation. A total of 100 ng of total RNA was reverse-transcribed using the PrimeScript RT Reagent Kit (Perfect Real Time) (TaKaRa). IE gene mRNA abundance was measured in the cDNA with SYBR Premix Ex Taq (Perfect real time) (TaKaRa) and TaKaRa Smart Cycler II (TaKaRa) quantitative real-time PCR technology. Real-time PCR was performed using the primer set listed in S1 Table. The thermal cycling program used was 95°C for 30 seconds, 95°C for 4 seconds, followed by 40 cycles of 60°C for 25 seconds. Each measurement was performed in triplicate, and the mean of the technical replicates was used. The measurements were performed in parallel with the quantitation of known amounts, ranging from 100 ag or 1 fg to 10 ng (at least 8 orders of magnitude), of IE gene expression cassettes to generate standard curves. The copy number of IE gene mRNA per 1×10^6^ cells was estimated using the standard curves. Log_2_-transformed copy numbers in the subsets of the dataset for each IE gene mRNA were separately fitted to a mixed linear model:

$$C_{ij}\;=\;G_{ij}+R_{ik}+\varepsilon_{ijk}$$

where *C*, *G*, *R*, and *ε* correspond to the log_2_-transformed copy number per 1×10^6^ cells, viral genotype, replicate, and residual, respectively. *G* is a fixed factor. *R* and *ε* are random factors. The mean estimate of the genotype was used as the modeled copy number. The *p*-value associated with the estimated genotype effect was corrected using the Benjamini-Hochberg False Discovery Rate [38] to obtain the *q*-value. The q-value was used to evaluate if the genotype effect was statistically significant (*α* = 0.1).

### Statistical modeling of the contributions of IE0, IE1, and the IE0–IE1 interaction to IE gene transcription

We estimated the contributions of IE0, IE1, and the interaction between them, as well as ME53, PE38, and others defined as “Rest” (any other unidentified factors, including host factors), by fitting the mixed general linear model [33,34]:

$$E_{ij}\sim {IE0}_{i}+{IE1}_{i}+{IE0:IE1}_{i}+{IE2}_{i}+{ME53}_{i}+{PE38}_{i}+{Rest}_{i}+\left(1|R_{j}\right)+\varepsilon_{ij}$$

where *E* represents the log_2_-transformed copy number of an IE gene (i = *ie2*, *me53*, and *pe38*) per 1×10^6^ cells. *IE0*, *IE1*, *IE0*:*IE1*, *IE2*, *ME53*, *PE38*, and *Rest* are fixed factors and represent the contributions of IE0, IE1, the IE0–IE1 interaction, IE2, ME53, PE38, and unidentified factors to the transcription of the IE gene, respectively. *R* represents the effect of the replicate and was a random factor.

## Results

### Study design

We designed a strategy to analyze, model, and test how AcMNPV IE gene expression was regulated (Fig. 1A). We first analyzed the *cis*- and *trans*-regulation of IE gene expression using a transient expression analysis. This analysis aimed to identify the regulatory functions of the IE genes on IE gene promoters in all pairwise combinations. By defining the viral components to be analyzed, we could collect information on the simple building blocks composing the IE gene regulatory network. The regulatory relationships observed in the systematic transient expression analysis were used to build a model for the AcMNPV IE gene regulatory network. The resulting network model was evaluated with network statistics to infer the properties governing IE gene expression.

After modeling, a reverse genetic approach using a bacmid system [36] was used to determine how IE gene expression was regulated in the context of infection. Each of the IE gene ORFs was replaced with an ampicillin resistance gene by homologous recombination, and sequences outside of the ORFs were left intact (Fig. 1D and S1B Fig.). Viral genomes lacking a single IE gene were generated to experimentally perturb the IE gene regulatory network. After the KO bacmid was transfected into Sf9 cells, the mRNA abundance of the remaining IE genes was measured by quantitative real-time PCR. By combining the results obtained in these analyses, we attempted to obtain insights into the regulatory mechanisms of IE gene expression at a network level.

A challenge in our study design was determining the time points that could effectively combine the results of the two assays. Due to the differences in the kinetics of viral gene expression and the sensitivity of the two assays, it was not possible to directly compare the results. To analyze the regulatory relationships between IE genes in the immediate early stage of infection, early time points were used to exclude the involvement of other viral genes, such as delayed early genes. However, results from preliminary experiments indicated that measuring reporter activities or mRNA expression levels at identical time points in the two assays did not have adequate resolution to reveal differences between control and test samples (data not shown).

Therefore, we prioritized sensitivity for detecting the possible differential expression of IE genes between control and test samples, and took measurements at 48 and 6 hours post-transfection (hpt) for the transient expression analysis and the reverse genetic approach, respectively. The time point 6 hpt was chosen for the reverse genetic approach to maximize differences in IE gene expression between mutants and the control, while minimizing the effects of other viral genes. Although we could not completely rule out the possibility that some delayed early and/or late viral genes, as well as other IE genes that were already expressed, may have participated in the regulation of IE gene expression at that time point, the most influential regulatory factors of IE gene expression were likely the IE genes, because they contain validated or plausible transcriptional regulatory domains and are expressed immediately after transfection. In addition, the regulatory activities of delayed early or late genes, which are controlled by the IE genes analyzed in this study, could be included in the regulatory roles of the IE genes analyzed.

Although 48 hpt for the transient expression analysis was considerably later than the immediate early stage of the infection, reporter activities at earlier time points were not high enough to obtain reliable readouts. Instead of a viral infection, this was a transfection with a reporter plasmid, which likely had a delayed onset of gene expression and could have had a delayed stimulation by the co-transfected plasmid carrying the IE gene. However, the reporter activities measured at the time point should reflect the activation or repression of the IE gene promoter by the IE gene in the early phase of infection.

For comparing the regulatory relationships between the IE genes across the two independent analyses, we limited the comparisons to qualitative regulatory relationships (i.e., positive, negative, and insignificant regulations), owing to the differences between the two assays, as described above. Positive regulation was defined in this study as a causal relationship, i.e., the presence of an IE gene led to a statistically significant increase in the expression of itself or another IE gene, compared to that of a corresponding control sample. The level of statistical significance was set to *q*-value < 0.1. Similarly, negative regulation was defined as a significant decrease in the expression of an IE gene, caused by the presence of another IE gene or itself. Note that the directions of differential expression for positive and negative regulations in the genetic analysis are opposite to the ones in the transient expression analysis. Quantitative comparisons of the contributions of the IE genes to expression were performed only within each analysis.

### Systematic transient expression analysis reveals that IE genes are generally transactivators

**Fig 2:**
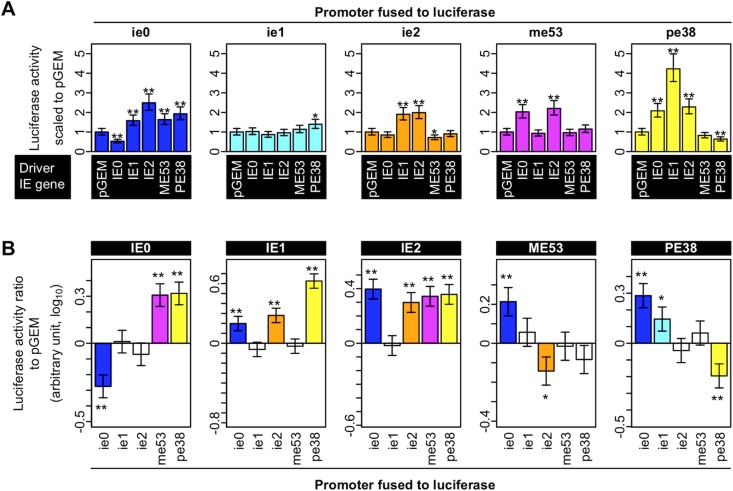
IE gene regulatory functions observed in the transient expression analysis. Luciferase activity was measured in cells 48 hours post-transfection in all pairwise combinations of the reporter and IE gene expression plasmids. (A) Comparison of the responsiveness of IE gene promoters to each co-expressed IE gene. The IE gene cassettes and the reporter plasmid used in each measurement are indicated in the columns below the panel and in the title of the panel, respectively. The estimated mean expression values of the IE gene cassettes are reported, after accounting for the value of the corresponding pGEM-transfected sample. The reported estimated mean values are the sums of the estimated effect of basal promoter activity and the effect of the tested gene on the promoter. Estimation of the values was performed using the mixed linear model. Error bars are the standard errors of the estimated means for the IE gene effects. Two sets of experiments, each containing a biological replicate, to estimate the random variation were done (These replicates were defined as “set” and “replicate” in Materials and Methods, respectively). (B) Comparison of the regulatory activities of the IE genes on each promoter. The reported values are the estimated gene effects. The IE gene cassette and the reporter plasmid used in each measurement are indicated in the title and in the columns of the panels, respectively. Asterisks indicate significant regulatory functions: *, *q*-value < 0.1; **, *q*-value < 0.05. The *q*-value is the *p*-value adjusted using the False Discovery Rate.

To map the regulatory relationships among AcMNPV IE genes, we tested for *cis*- and *trans*-regulation between all pairwise combinations of IE genes (Fig. 2). A reporter plasmid carrying an IE gene promoter fused with firefly luciferase (indicated as the title of the plot in Fig. 2A and in the columns in Fig. 2B) was co-transfected with a control plasmid (pGEM-Teasy) or a plasmid that expressed an IE gene (indicated in the columns of Fig. 2A as the “Driver IE gene” and in the title of the plot of Fig. 2B). Luciferase activity was measured at 48 hpt, and the measured values were fit to a mixed linear model. Modeled measured values were subjected to a statistical analysis to detect significant activation or repression of each promoter by each IE gene. Significant changes were observed in 15 out of the 25 examined IE gene–IE promoter regulatory relationships (Fig. 2; *q*-value < 0.1). Eleven were transactivation regulations, suggesting that IE genes function mostly as transactivators. The *ie0* promoter was activated by all IE genes, except for IE0 itself, which repressed it. The *ie1* promoter was only activated weakly by PE38. The *ie2* promoter was activated by IE2 and IE1, and repressed by ME53. The pattern of regulation of the *pe38* promoter was similar to that of *ie0*. Most of the IE genes activated the promoters of *ie0* and *pe38*, and negative autoregulation of the two genes was observed. In contrast to the other IE promoters, the *me53* promoter was not activated by IE1, but by IE0 and IE2. Although the regulatory relationships between IE1 and the *ie1* and *ie0* promoter in our study were inconsistent with those in a previous study [12], it was not feasible to identify a factor(s) causing the inconsistency. In addition, we aimed to model the regulatory network in order to understand how IE gene expression was controlled at a system level. Therefore, we focused on a systematic analysis of IE gene regulation, rather than a detailed investigation of each IE gene’s function, an investigation that could have, perhaps, explained these inconsistencies.

### Properties of the IE gene transcriptional regulatory network

**Fig 3:**
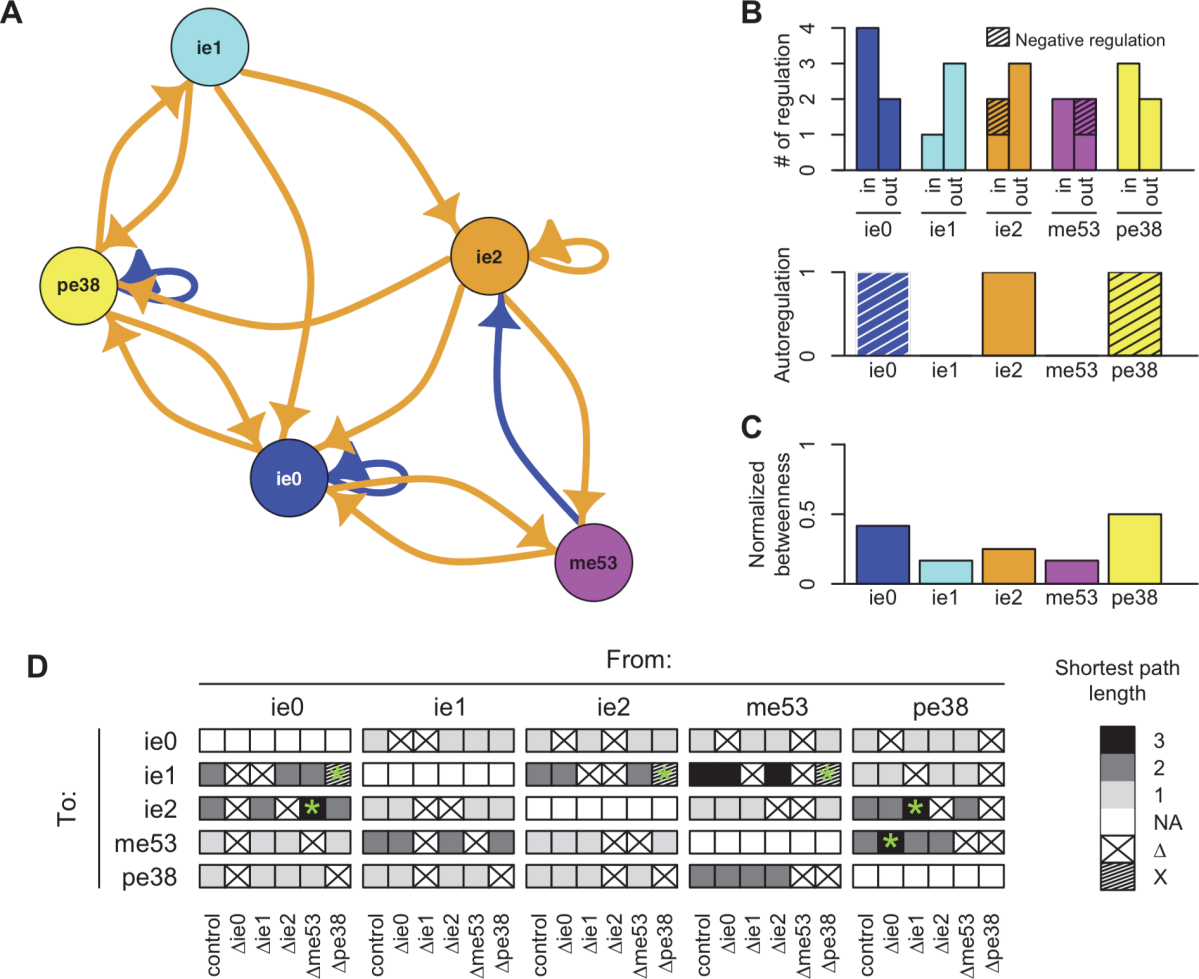
Network analysis of IE gene regulatory functions. (A) A network graph of the IE gene regulatory functions observed in the transient expression analysis. Orange and blue links indicate positive and negative regulatory functions, respectively. The width of the links corresponds proportionally to the absolute values of the ratio values shown in Fig. 2B, representing the strength of the regulatory function. (B) The number of links to each IE gene in our network model. “In” and “out” represent incoming links to and outgoing links from the IE genes as indicated below. (C) Normalized betweenness of IE genes in our IE gene network model. (D) Possible path redundancy in the IE gene regulatory network. The simulation of genetic perturbations was performed by calculating the shortest path length following removal of one IE gene from the model. Crossed cells indicate combinations of input and output IE genes in which the shortest path length could not be computed. Shaded cells indicate the isolation of the output IE gene due to the removal of IE genes connected with it.

We compiled the detected regulatory relationships and visualized them as a network graph (Fig. 3A). The IE genes and their regulatory relationships were represented as nodes and links, respectively. Hereafter, this network is designated “our IE gene network model” because it was compiled solely based on our results.

### Possible roles for each IE gene in the network

We examined the links representing the regulation of each IE gene. The number of incoming and outgoing links is shown in Fig. 3B. *ie1* had only one incoming link and 3 outgoing links, which may suggest that IE1 is a primary regulator that directs the expression of other IE genes. *ie2* also had more outgoing links than incoming links, apart from its autoregulation. *ie2* was an activator for all the IE gene promoters, except for *ie1*. *me53* was the only gene with a negative outgoing link under our experimental conditions, indicating that *me53* may have particularly important regulatory functions. In contrast, *ie0* and *pe38* had many incoming links. *ie0* was the common target for all IE genes, and both *ie0* and *pe38* were negatively autoregulated.

### High connectivity among IE genes in our IE gene network model

The lengths of the shortest paths (i.e., the minimum number of steps for potential sequential regulation between IE genes) were examined to evaluate the global organization of the network. The maximum length of the shortest path was 3, which was the path between *ie1* and *me53*. This indicated that *ie1* and *me53* were relatively independent within the IE network. Normalized betweenness, the frequency with which a node exists on the shortest paths between all combinations of two nodes, also revealed that *ie1* and *me53* did not contribute to the interconnectivity of the network (Fig. 3C). On the other hand, *ie0* and *pe38* appeared to be the hubs. Approximately half of the shortest paths were through these two genes. The average length of the shortest paths between IE genes was 1.45, which suggested that most IE gene pairs may be directly connected or have only one gene between them. It also indicates that the IE gene network was well interconnected.

### Redundant activation paths between IE genes

Our IE gene network model had high connectivity, and each IE gene had more than one incoming link (2.2 on average). We extended the search for redundant activation paths from one IE gene to another, paths consisting of up to three genes (feed-forward loop). Feed-forward loops with *ie1* as an input gene were frequently observed (S3 Fig.). To evaluate the overall redundancy of paths between IE genes, we simulated perturbations by removing one gene from the IE gene regulatory network model and calculating the resultant lengths of the shortest paths between all combinations of IE gene pairs. Only 10% (6 out of 60) of the simulated perturbations changed the length of the shortest path.

### Robust control of IE gene expression against single IE gene deletions

**Fig 4:**
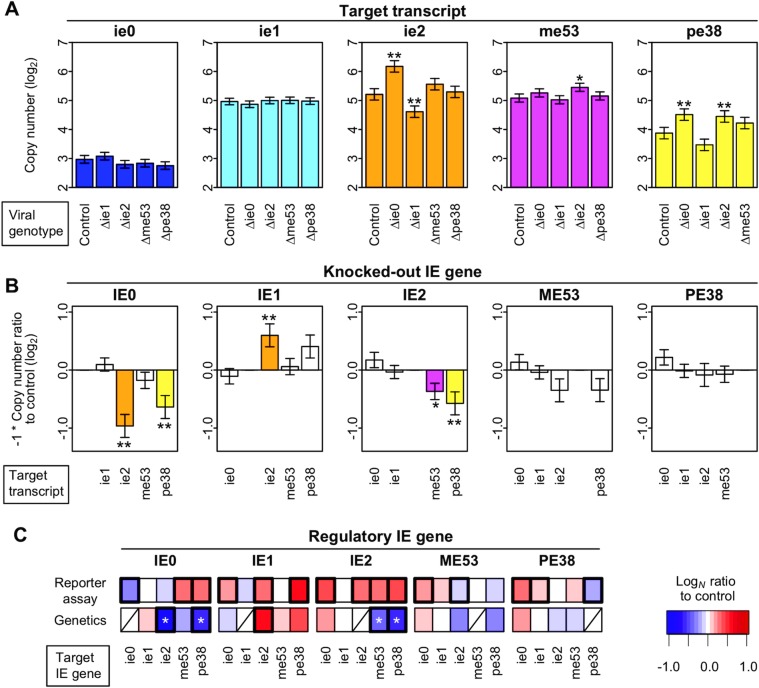
IE gene regulatory functions observed in the reverse genetic analysis. The abundance of IE mRNA in cells transfected with each IE gene knockout virus 6 hours post-transfection was quantified using real-time PCR. (A) Comparison of IE gene expression level in each knockout virus. The estimated mean copy numbers of each transcript (indicated by the label on the panel) with each genotype (indicated by the columns below the panel) represented as heights of the bars (the vertical axis). The values are the sums of the estimated steady-state expression level in the control virus and the estimated genotype effect of the knockout virus. Error bars are standard errors of the estimated copy numbers. These values were estimated by fitting a mixed linear model. Asterisks indicate significant regulatory functions: *, *q*-value < 0.1; **, *q*-value < 0.05. The *q*-value is the *p*-value adjusted using the False Discovery Rate. The reported values are calculated from three technical replicates and three biological replicates. (B) Comparison of IE gene regulatory activity on IE gene transcription. The estimated genotype effect on the copy number of each transcript (indicated by the columns below the panel) used in (A) multiplied by -1, represented as the estimated regulatory activity of the wild-type viral gene. The vertical axis indicates quantity of regulatory activity and direction of the regulation. (C) Comparison of IE gene regulatory functions in the genetic analysis using knockout viruses to those in the transient expression analysis using the reporter assay. The values and statistics are shown in Figs. 2B and 4B for the “Reporter assay” and “Genetics”, respectively. The logarithmic bases of the values for the transient expression analysis and the genetic analysis are 10 and 2, respectively. Shaded cells indicate transcripts not measured because the genes were removed from the genome. Bold boundaries indicate significant regulatory relationships (*q*-value < 0.1). Asterisks indicate a genotype–phenotype paradox, in which a perturbation to a gene with an activator function or no regulatory function in the transient expression analysis resulted in enhanced target gene expression in the genetic analysis.

We experimentally perturbed the IE gene regulatory network using IE gene knockout viruses, in which an IE gene was removed from the genome, and quantified IE gene mRNA levels during infection. Bacmids with a single IE gene removed were constructed for all the IE genes and were used for the transfection of Sf9 cells. IE gene mRNA levels at 6 hpt were measured using quantitative real-time PCR (Fig. 4A). *ie0* and *ie1* mRNA levels were not significantly changed by knocking out any one of the IE genes. *ie2* mRNA levels were up- or down-regulated during infection with *ie0* or *ie1* KO virus, respectively. *me53* mRNA levels were changed only when *ie2* was knocked out. Knockout of *ie0* or *ie2* caused a small, but statistically significant, increase in *pe38* mRNA levels (*q* = 0.036 and 0.047, respectively), whereas knockout of *ie1* caused a marginal reduction (*q* = 0.16).

These results were not readily predictable solely from the results of the transient expression analysis, and two inconsistencies were observed. First, IE gene expression was tolerant to most genetic perturbations of the genes. Although IE genes were identified as transactivators in the transient expression analysis, the network maintained expression levels during the early phase of infection, even when a transactivator IE gene was knocked out. If the contribution of each IE gene to the expression of the target IE genes was as substantial as it was in the transient expression analysis, the expression of the target IE genes should have changed significantly when each IE gene was perturbed. Second, *ie0* and *ie2* KO viruses showed paradoxical phenotypes. The expression of *pe38* in the *ie0* KO virus, as well as *me53* and *pe38* in the *ie2* KO virus, was enhanced rather than reduced by perturbations to their transactivator genes, *ie0* and *ie2*, respectively. If the phenotypes of IE gene KO viruses are interpreted as being opposite to the functions of the gene in the wild-type virus, they would be considered repressors for the target genes (Fig. 4B). The regulatory relationships observed in the transient expression analysis, which examined the regulation of one IE gene promoter by one IE gene, did not explain the paradoxical expression phenotypes observed in the genetic analysis, which investigated the regulatory functions of IE genes through the interactions between them.

### Quantitative nature of the IE0–IE1 interaction

We hypothesized that the possible collective behavior of the IE genes generated more complex, interactive regulatory mechanisms during infection. To test this hypothesis, we chose IE0 and IE1 for a more detailed examination for the following reasons: (1) The enhanced expression of *ie2* and *pe38* in the *ie0* KO virus was inconsistent with the results of the transient expression analysis, suggesting that the function of IE0 changed in the presence of other IE genes. (2) Although the *ie2* KO virus similarly showed IE gene expression paradoxical to the regulatory functions observed in the transient expression assay, the combination of IE0 and IE1 was preferred because of their stable expression. Both *ie0* and *ie1* expression levels were highly stable in all KO viruses, whereas the expression of *ie2* varied (Fig. 4A). It was conceivable that IE0–IE1 interactions have consistently occurred in our genetic experiments, because both *ie0* and *ie1* mRNA levels were stable in cells transfected with any single KO virus, except for ones lacking *ie0* or *ie1*. (3) IE0 and IE1 interact and form heterodimers [40]. (4) Negative effects on IE gene transcription were greater in the *ie0* KO virus than in the *ie2* KO virus (Fig. 4C).

**Fig 5:**
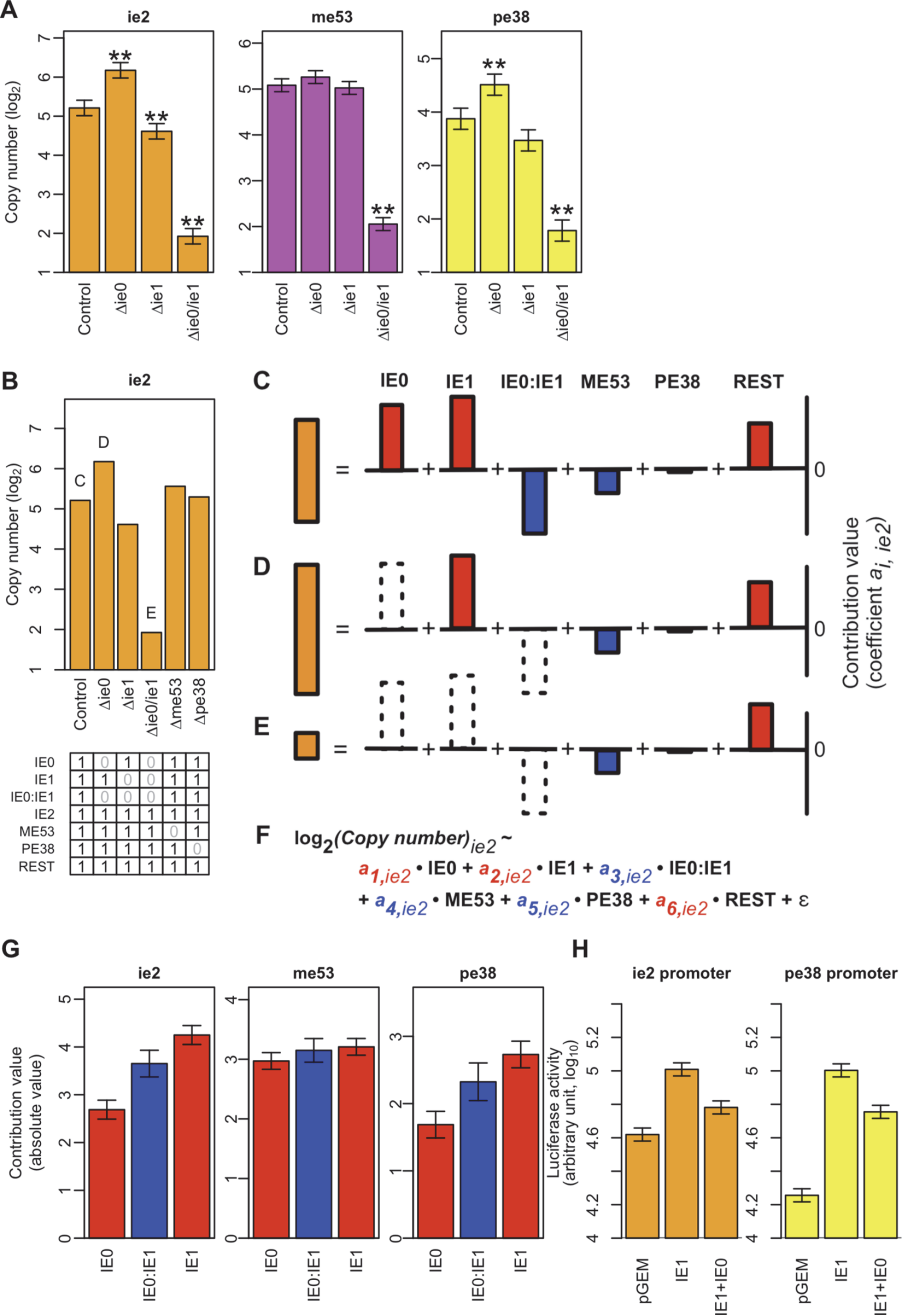
The IE0–IE1 interaction contributed to the expression of other IE genes in a distinct manner. (A) IE mRNA abundance in *ie0*, *ie1* single, and *ie0*/*ie1* double knockout viruses 6 hours post-transfection. Error bars are standard errors of the estimated means. Asterisks indicate significant regulatory functions: **, *q*-value < 0.05. The *q*-value is the *p*-value adjusted using the False Discovery Rate. (B) Values used for model fitting to estimate the contribution of IE0 alone, IE1 alone, the IE0–IE1 interaction to the expression of other IE genes, and the rest. Top, real-time qPCR data for the expression of *ie2*, as an example. The mean expression levels of *ie2* were estimated using a linear model, with the viral genotypes indicated below. Letters C, D, and E in Fig. 5C, D, and E refer to the letters C (control), D (Δie0), and E (Δie0/ie1) above the columns in Fig. 5B. Bottom, the values assigned to the variables in the model for the single gene and interaction effects. The values (0 or 1) indicate presence or absence of the genes or interaction (indicated by the row label) in the genotype (indicated by the column below the matrix). The values were used to fit the linear model shown in F. (C, D, F) Schematic representations of the decomposition of the contribution of single genes and the IE0–IE1 interaction to IE gene expression. Red and blue bars indicate the positive and negative contribution of each gene or interaction to IE gene expression, respectively. (C) The wild-type virus had a complete set of genes. (D) The *ie0* knockout virus lacked the contribution of IE0 to IE gene expression, and naturally lacked the IE0–IE1 interaction. (E) *ie0*/*ie1* knockout virus lacks IE0, IE1, and the IE0–IE1 interaction. (F) The linear model used to estimate the contribution of single gene and interaction effects. The values (0 or 1) shown in B were assigned to the gene and interaction variables (IE0, IE1, IE0:IE1, ME53, PE38, and REST). (G) Estimated contribution of IE0, IE1, and the IE0–IE1 interaction to the other IE genes. Note that the height of the bars indicates the absolute values of the estimated contributions. Red and blue bars indicate the positive and negative contribution of each gene or interaction to IE gene expression, respectively. Error bars are standard errors of the estimated means. (H) Transient expression analysis to confirm the effects of the IE0–IE1 interaction on the expression of *ie2* and *pe38*. The *ie2* or *pe38* promoter plasmids were co-transfected with the plasmid(s) expressing the IE gene(s), indicated as “Driver IE gene” in the column. The estimated mean values of the IE gene cassettes were adjusted by the value of the corresponding pGEM-transfected samples. The reported estimated mean values are the sums of the estimated effect size of the basal promoter activity and the IE gene on the promoter. Estimation of the values was performed using the mixed linear model. Error bars are standard errors of the estimated means for the IE gene effects. Two sets of experiments to estimate the random variation in a biological replicate were done (These replicates were defined as “set” and “replicate” in Materials and Methods, respectively). Luciferase activity was measured in transfected cells at 48 hours post-transfection. Error bars are standard errors of the estimated means.

We examined how the IE0–IE1 interaction contributed to the transcription of other IE genes, using simultaneous perturbations of both *ie0* and *ie1* in the viral genome. When both *ie0* and *ie1* were simultaneously knocked out, the expression of *ie2*, *me53*, and *pe38* was markedly decreased at 6 hpt (Fig. 5A). Even though regulation of *me53* by IE0 or IE1 was not detected in either the transient expression analysis or the genetic analysis, the importance of IE0 and IE1 in regulating *me53* expression during infection became evident upon simultaneous perturbation. It should be noted that the term ‘IE0–IE1 interaction’ includes interactions with other IE genes. For example, an IE0–IE1–IE2 interaction and higher-order interactions including IE0 and IE1 were included in this term for the purpose of the experimental design. Similarly, the functions of IE0 alone and IE1 alone, analyzed using *ie0* KO and *ie1* KO viruses, respectively, also contained interactions with other IE genes. Nevertheless, the IE0–IE1 interaction with its current definition, not IE0 or IE1 alone, regulated the expression of other IE genes.

We then analyzed the quantitative contribution of IE0, IE1, and the IE0–IE1 interaction to IE gene transcription using statistical modeling. We used a linear model to separate and estimate the contribution of the above factors and the interaction [33] (Fig. 5B-F). We fit a model consisting of IE0, IE1, and the IE0–IE1 interaction to quantitative real-time PCR data (Fig. 5G; estimates for all variables are shown in Table 1). The estimated coefficients for the explanatory variables were interpreted as quantitative measures of their contribution to IE gene expression.

**Table 1:** Summary of statistical modeling of contribution of IE genes, IE0:IE1 interaction, and remaining factors.

Target	Factor	Estimate	S.E.	*q*-value
**ie2**	IE0	2.688	0.198	0.00000
	IE1	4.250	0.198	0.00000
	IE0:IE1	-3.652	0.279	0.00000
	ME53	-0.350	0.198	0.12500
	PE38	-0.085	0.198	0.67600
	REST*	2.360	0.370	0.00012
**me53**	IE0	2.971	0.140	0.00000
	IE1	3.208	0.140	0.00000
	IE0:IE1	-3.149	0.198	0.00000
	IE2	-0.369	0.140	0.03230
	PE38	-0.072	0.140	0.65300
	REST*	2.495	0.264	0.00001
**pe38**	IE0	1.687	0.198	0.00001
	IE1	2.730	0.198	0.00000
	IE0:IE1	-2.324	0.280	0.00002
	IE2	-0.574	0.198	0.02190
	ME53	-0.346	0.198	0.12500
	REST*	2.703	0.372	0.00004

* Remaining factors such as host factors contributing to IE gene expression.

The model inferred that both IE0 and IE1 positively contributed to other genes’ expression, and the estimated IE0 contribution was inconsistent with the interpretation of the results from the genetic experiments. On the other hand, the contribution of the IE0–IE1 interaction to the expression of the other IE genes was estimated to be negative. The absolute value of the negative contribution of the interaction between IE0 and IE1 to *ie2* and *pe38* transcription was larger than that of the positive contribution of IE0. Thus, the enhanced transcription of *ie2* and *pe38* observed in the *ie0* KO bacmid–transfected cells could be explained as a phenotype that resulted from the differential contribution of IE0 and the IE0–IE1 interaction.

To confirm the negative effects of the IE0–IE1 interaction, we performed a transient expression analysis with a single transfection or co-transfection of plasmids carrying *ie0* and *ie1* (Fig. 5H). Consistent with the combinatorial genetic analysis, the co-transfection of plasmids carrying *ie0* and *ie1* reduced the activity of the reporter, relative to the activity of a plasmid carrying only *ie1*.

## Discussion

This study revealed the robust control of IE gene expression during the early phase of infection and the importance of the interactions between these IE genes, suggesting possible network-level regulations. The regulatory functions of the IE genes differed when they were analyzed in isolation or as a component of the gene regulatory network. The contrasting results obtained from the transient expression and genetic analyses highlighted that the functions of the IE genes change upon interacting with other IE genes. In addition, network-level regulations appeared to play critical roles in robustly controlling the IE gene expression early in infection. The regulatory relationships observed in the transient expression analysis, as a network, suggested collective behaviors of multiple IE genes (Fig. 3). Based on our IE gene regulatory network model, we hypothesized that at least three mechanisms might confer robustness to the IE gene expression program: (1) The exploitation of the host transcriptional machinery to decrease dependency on other viral genes; (2) redundant stimulation by multiple viral genes; (3) redundant regulatory paths between input and output IE genes.

### Exploitation of the host transcriptional machinery to decrease dependency on other viral genes

Each IE gene appeared to be regulated in a distinct manner. A mechanism that could confer robustness to the IE gene expression program against perturbations of viral genes would be to exploit the host transcriptional machinery to decrease dependency on viral factors. The regulation of *ie1* expression fits this hypothesis. In the transient expression analysis, only PE38 showed weak activation of the *ie1* promoter. In the genetic analysis, the expression of *ie1* did not significantly change in any of the IE gene KO viruses, suggesting the reliance on a host factor. A drawback of this strategy is that IE gene regulation would become sensitive to the absence of the host factors required for sufficient expression [41], a condition that may be overcome by positive autoregulation. However, we could not detect the autoregulation of *ie1* under our experimental conditions, which may have induced sufficiently strong *ie1* expression to mask, or avoid triggering, *ie1* positive autoregulation [12,42,43].

### Redundant stimulation of IE gene promoters by multiple viral genes

The second possible mechanism for robustly controlling expression of an IE gene would be to have multiple IE genes functioning as expression regulators, with not all of them required simultaneously. Comparison of the results obtained from the transient expression analysis and those from the genetic analysis suggests that the expression of *ie0* was regulated using this logic. Given that the host factor–driven basal expression levels of *ie1*, *ie2*, *me53*, and *pe38* were sufficient to activate the expression of *ie0*, as shown in the transient expression analysis, it is likely that *ie0* activation by multiple IE genes occurs during infection. The genetic analysis revealed that, at most, three IE genes in various combinations are sufficient to accomplish an *ie0* expression level similar to that of the wild-type virus. Whereas this type of regulation appeared to be tolerant to the lack of a viral gene(s), it may potentially result in over-activation by redundantly existing activators. The expression of *ie0* was found to be negatively autoregulated, which could potentially prevent over-activation. This strategy could produce a rapid, initial rise in expression levels, followed by a subsequent locking into steady-state levels using negative autoregulation [44]. The expression of an immediate early gene of the herpesvirus exhibited these accelerator properties [45]; they conferred a fitness advantage to the virus over the amplifier-type of regulation that results in overexpression of the IE gene. Although no empirical evidence regarding the toxicity of *ie0* overexpression is available, it has been demonstrated in a transient replication assay that LdMNPV IE0 stimulates replication of a reporter plasmid containing the LdMNPV hr4 replication origin [16], and baculoviral DNA replication induces apoptosis [46]. Excess stimulation of baculoviral DNA replication caused by overexpression of *ie0* may decrease viral fitness by increasing apoptotic cell death in the host.

### Extensive connectivity creates multiple paths between input and output IE genes

In addition to redundant direct connections between IE genes (the average number of incoming links to an IE gene was 2.2), indirect connections through one or more intervening genes also appeared to be redundant. Feed-forward loops composed of three genes and with *ie1* or *ie2* as the input gene appeared to prevail in our IE gene regulatory network model. Although we cannot rule out the possibility that multiple paths between input and output IE genes may be involved in fine-tuning the expression of the output genes, our simulation and genetic analysis indicated that multiple paths that share the same input and output genes could provide suboptimal paths for output gene expression, even when the optimal path is perturbed. Extensive direct and indirect connectivity in the network may create a means to maintain the expression levels of IE genes against perturbations.

### Contribution of other viral genes

We have tested the 5 IE genes for their regulatory relationships but there might be other viral genes regulating the IE gene expression at the time point. Other IE genes such as he65 as well as delayed early and/or late genes expressed by 6 hpt may have contributed to the IE gene expression program. Feedbacks from delayed early genes and/or late genes to the IE genes have not been studied but might exist. However, in our current understanding of baculoviral gene expression, the five IE genes are major regulators of expression of the baculoviral genes including he65, delayed early genes, and late genes. In addition, major contribution of ie0 and ie1 to the IE gene expression was evident in the genetic analysis using Δ*ie0*/*ie1* bacmid (Fig. 5A). Although we cannot rule out possible contribution of viral genes that were not tested in this study to the IE gene expression, we assume that regulatory activity of the genes could be considered as a part of the regulatory activity of the tested IE genes or their contribution to the IE gene expression is relatively small.

### Significance of viral gene interactions in the regulation of IE genes

Our analyses revealed unpredicted aspects of IE gene expression, as well as robust control of expression in knockout viruses. The deletion of *ie1* led to a reduction in *ie2* transcription, which was expected because IE1 is the only activator of *ie2* among the IE genes analyzed in this study (Figs. 2B and 4B). However, the deletion of *ie0* (activator of *pe38*, *ie0*, and *me53*) and *ie2* (activator of *pe38* and *ie2)* enhanced the expression of the target IE genes. These phenotypes disagreed with the regulatory functions identified by the transient expression analysis, which suggested that they have regulatory functions that were not evident when each gene was analyzed in isolation. Statistical analyses, in combination with combinatorial genetics, revealed that the IE0–IE1 interaction negatively regulated the expression of *ie2*, *me53*, and *pe38* (Fig. 5G). We hypothesize that the negative regulation by the IE0–IE1 interaction may be compensatory and might maintain expression at an optimal level.

The IE genes appear to have evolved towards an optimized trade-off between the rapid induction of IE gene expression and the suppression of expression at unnecessarily high levels. The IE genes must be rapidly and strongly induced upon infection in order for the virus to immediately execute the viral transcriptional program and to manipulate the host environment. For this purpose, the IE genes are likely to have evolved as transactivators. At the same time, the overexpression of viral genes by transactivators should also be prevented, to avoid unnecessarily high or cytotoxic levels. Therefore, repression following the successful increase of viral gene expression may be necessary for an effective viral transcriptional program.

One possible mechanism for the repression of IE gene expression could be the concentration of an IE gene product triggering a regulatory mechanism. IE1 was previously shown to regulate the expression of *ie0*, *ie2*, and *pe38* in a concentration-dependent manner by binding to an IE1-binding motif in the promoters [29]. Another mechanism could use the interactions between IE genes. When most or all of the IE genes are being transcribed efficiently, there would be enough gene products to interact with one another, signaling a proper execution of the viral transcription program.

### The need for technologies that can further study the viral gene regulatory network

We used two complementary analyses to analyze the regulatory functions of IE genes: a transient expression analysis to examine the regulatory functions of the defined viral factors and a genetic analysis to investigate the regulatory functions within the context of infection. Both were necessary to gain insight into the properties of IE gene expression regulation and to reveal the importance of interaction between IE genes. However, contextual differences between two independent analyses may have caused inconsistencies between the results obtained in the analyses and, in addition, would hamper obtaining more validated gene regulatory network model in the future studies. We cannot rule out possibility that differences in the quantitation methods, and accordingly, time points and promoter sequences between the analyses caused the inconsistencies between results. Two technical problems must be addressed to further analyze the baculovirus gene regulatory network.

First, development of methods more sensitive to quantify IE gene expression is required for measuring regulatory activity of an IE gene at same time points, preferably time points when only IE genes are expressed, in the two analyses. Late gene expression at some extent was observed 6 hpt (S4 Fig.) and may have affected the results in the genetic analysis. In both analyses, measuring regulatory activity of an IE gene at earlier time points than 6 hpt was difficult due to insufficient quantitation limits in the conventional methods we used in this study. Follow-up experiments using more sensitive quantitation methods, such as digital PCR, and/or more biological replicates to increase statistical power will be required to definitively validate our conclusions.

Second, more efficient and extensive genome engineering of baculoviral genomes is required. Partial overlaps between the viral genetic elements may also have caused inconsistencies between the results. We arbitrarily defined promoter sequences as ~500 bases upstream of the IE gene ORFs based on previous studies [47,48] and created knock out bacmids by replacing entire ORFs with the antibiotic resistance gene to produce complete loss-of-function mutants except for Δie0. Overlaps or proximity between some viral genes may have caused inconsistencies between the results. For instance, the distance between *me53* and *ie0* ORFs is less than 500bp so that removing *me53* or *ie0* ORF from the bacmid results in truncation of the 500bp upstream sequence that was used in the transient expression analysis. To overcome this problem, refactoring the viral genome to make each functional element independent is necessary [49]. Moreover, our study revealed the importance of interaction between the viral genes. In order to analyze the contributions of higher-order interactions among the tested IE genes, it is necessary to generate all 32 genotypes (5 single mutants, 10 double mutants, 10 triple mutants, 5 quadruple mutants, 1 quintuple mutant, and the wild type). Therefore, to refactor viral functional elements and to extend our combinatorial genetic approach, the development of fast and high-throughput genome manipulation technologies, such as genome assemblies of the desired genotypes from DNA fragments and/or the simultaneous introduction of point mutations to the genome [50,51], are needed.

## Concluding Remarks

We have systematically collected information on regulatory relationships among the five IE genes, *ie0*, *ie1*, *ie2*, *me53*, and *pe38* using transient expression analysis and have built a gene regulatory network model. Expression of the IE genes at early phase of infection was robustly maintained against perturbations to single genes as inferred from the network model. On the other hand, inconsistencies of regulatory function of IE genes were found between results of the transient expression and the genetic analyses: IE0 and IE2 appeared to have opposite functions. The focused experiments with a combinatorial genetic approach using *ie0*, *ie1* single, *ie0*/*ie1* double mutants and the wild type, together with statistical modeling, revealed that IE0-IE1 interaction quantitatively affected the regulation of the IE gene expression. This study provided evidence of previously unappreciated regulation of baculoviral gene expression, revealed limitations of current experimental approaches, and suggested strategies to obtain better understanding of baculoviral gene regulation in future studies.

## Supporting Information

S1 Fig Generation of AcMNPV IE gene knockout bacmids. (A) The AcMNPV IE gene locus and scheme for generating knockout bacmids. *Polh*: *polyhedrin* gene, *Gm* ^*r*^: gentamycin resistance gene, *Amp* ^*r*^: ampicillin resistance gene. (B) Schematic representation of gene targeting via homologous recombination.
(TIF)

S2 Fig Confirmation of the IE KO bacmids by PCR analysis. Targeted regions were amplified using PCR. (A) AcP^+^ was a positive control (upper panel) and each IE gene knockout bacmid is in the lower panel. (B) *Polyhedrin* region. PCR products are shown in the ethidium bromide–stained agarose gels, and the arrowhead indicates the size of the products. (C) Upstream and downstream regions of each knockout were amplified using primers (S1 Table). M: λ*Eco*T14 I marker, 1: Δ*me53*, 2: Δ*ie0*, 3: Δ*ie1*, 4: Δ*ie2*, 5: Δ*pe38*.
(TIF)

S3 Fig Possible activation paths consisting of redundant feed-forward loops in the AcMNPV IE gene regulatory network. Feed-forward loops consisting of three genes and sharing the input (top) and output (bottom) IE genes were extracted from our network model (Fig. 3A). Links are the regulatory relationships detected in the transient expression analysis (Fig. 2A, B).
(TIF)

S4 Fig Expression of late genes *Orf141* and *Orf146* in Sf-9 6 hours post-transfection. The abundance of mRNA of *Orf141* or *Orf146* in cells transfected with control, *ie0*, or *ie1* gene knockout virus 6 hours post-transfection was quantified using real-time PCR. The estimated mean copy numbers of ie0, Orf141, or Orf146 transcript in cells transfected with bacmids indicated by the columns below the panel are shown. Expression of ie0 transcript was measured as a positive control for that of the late genes. The values are the sums of the estimated steady-state expression level in the control virus and the estimated genotype effect of the knockout virus. Error bars are standard errors of the estimated copy numbers. These values were estimated by fitting a mixed linear model. The asterisk (*) indicates significant regulatory functions: *p*-value < 0.05 in comparison to the control. The reported values are calculated from three technical replicates and three biological replicates.
(TIF)

S1 Table List of nucleotide sequences for primers used in this study. Restriction enzyme sites are underlined.
(DOCX)
