# Supplementary figures and images for: Tightly Regulated Expression of Autographa californica Multicapsid Nucleopolyhedrovirus Immediate Early Genes Emerges from Their Interactions and Possible Collective Behaviors
Source: PLoS One. 2015 Mar 27;10(3):e0119580. doi: 10.1371/journal.pone.0119580 (PMC4376880; doi:10.1371/journal.pone.0119580)

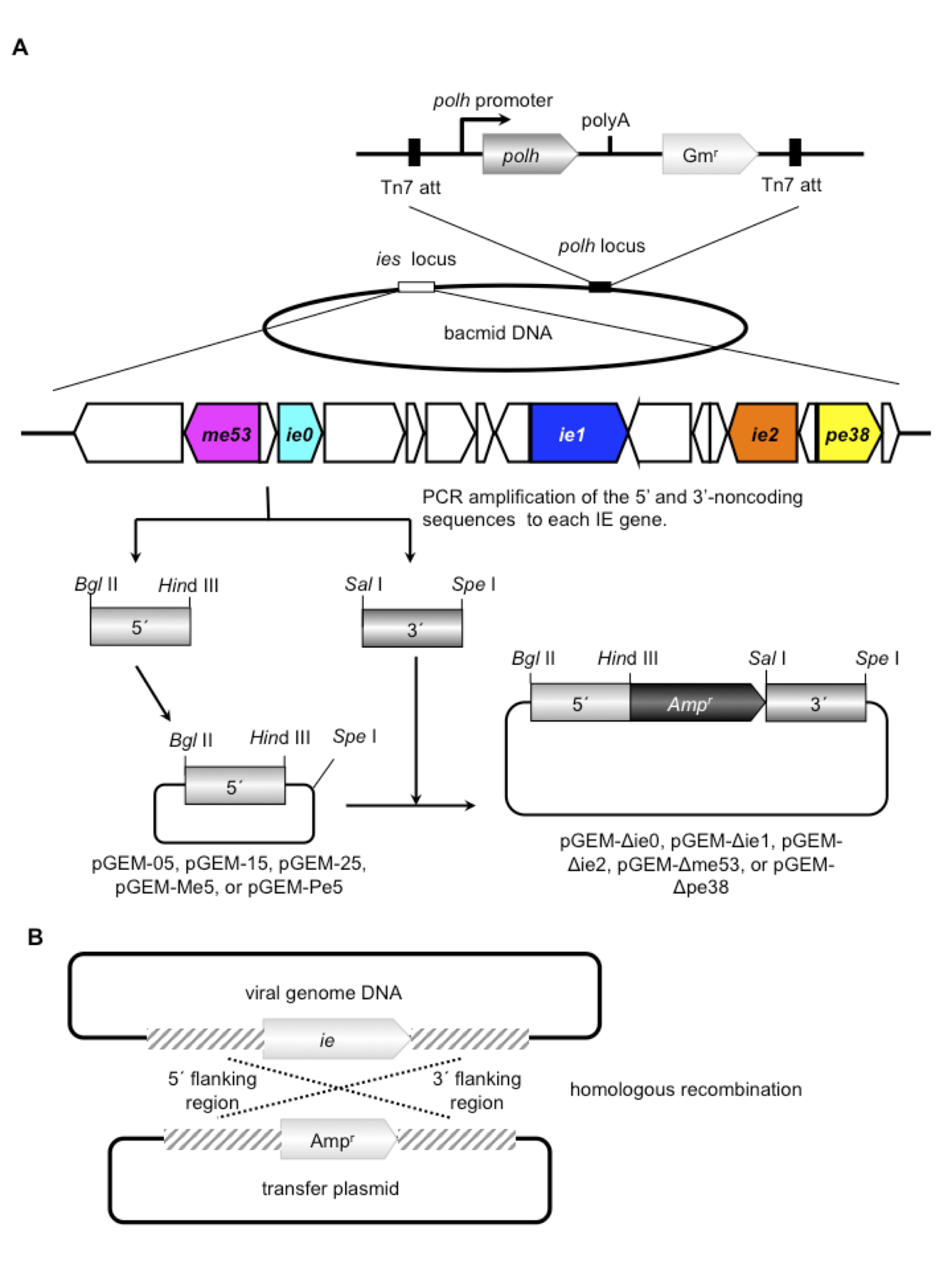

Supplement: S1 Fig — (A) The AcMNPV IE gene locus and scheme for generating knockout bacmids. Polh: polyhedrin gene, Gm r: gentamycin resistance gene, Amp r: ampicillin resistance gene. (B) Schematic representation of gene targeting via homologous recombination. (TIF) [file pone.0119580.s001.tif]

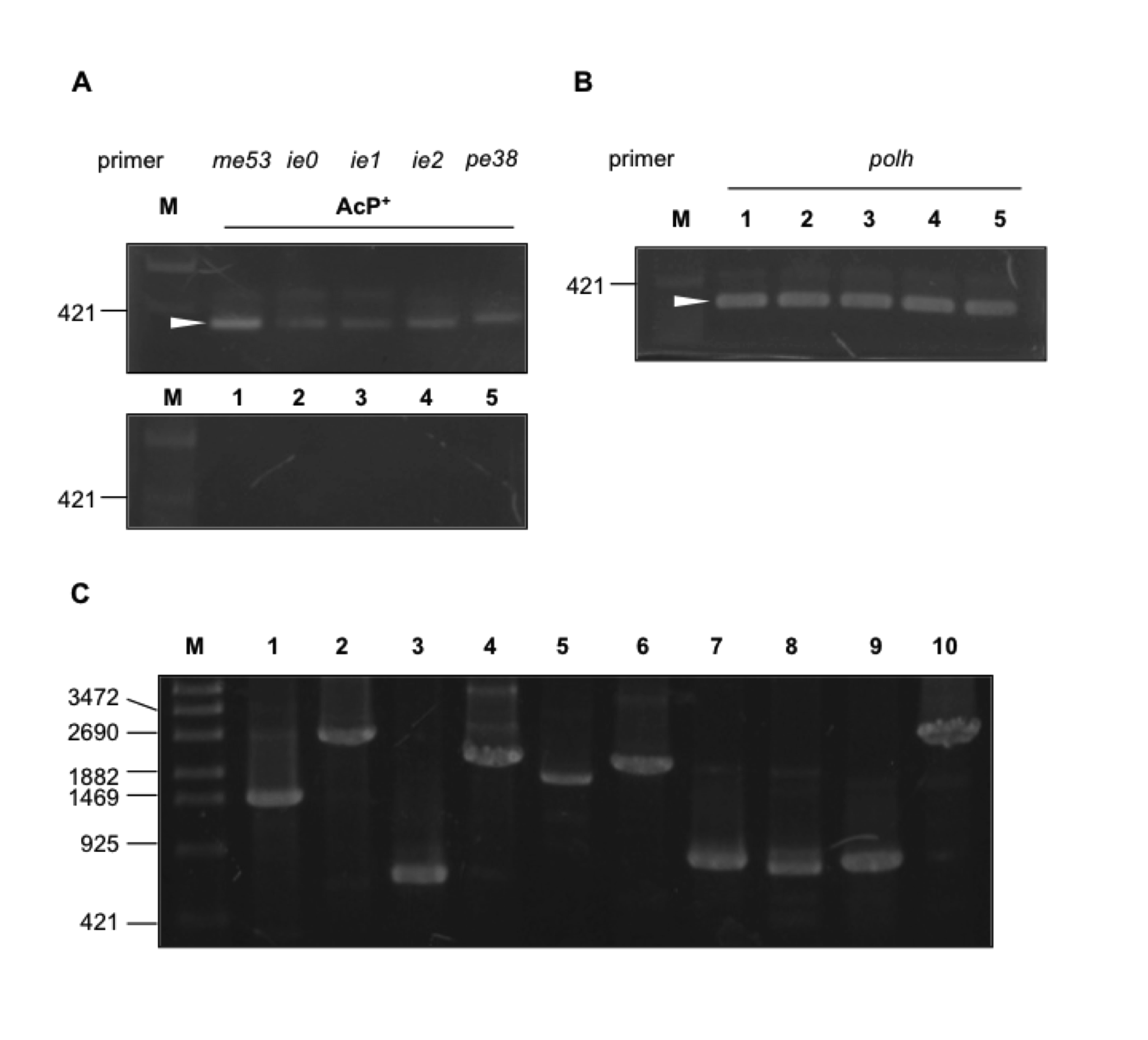

Supplement: S2 Fig — Targeted regions were amplified using PCR. (A) AcP+ was a positive control (upper panel) and each IE gene knockout bacmid is in the lower panel. (B) Polyhedrin region. PCR products are shown in the ethidium bromide–stained agarose gels, and the arrowhead indicates the size of the products. (C) Upstream and downstream regions of each knockout were amplified using primers (S1 Table). M: λEcoT14 I marker, 1: Δme53, 2: Δie0, 3: Δie1, 4: Δie2, 5: Δpe38. (TIF) [file pone.0119580.s002.tif]

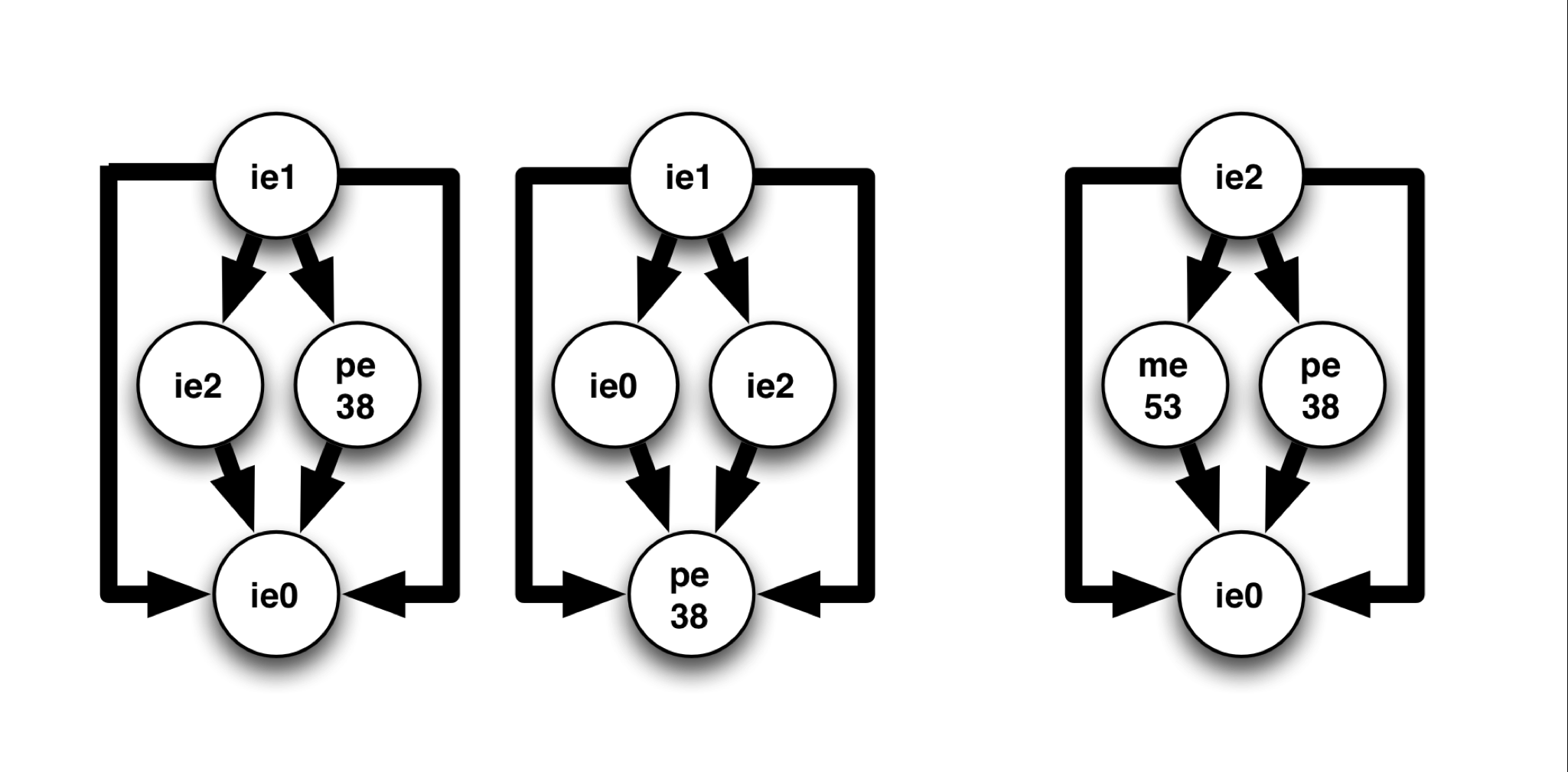

Supplement: S3 Fig — Feed-forward loops consisting of three genes and sharing the input (top) and output (bottom) IE genes were extracted from our network model (Fig. 3A). Links are the regulatory relationships detected in the transient expression analysis (Fig. 2A, B). (TIF) [file pone.0119580.s003.tif]

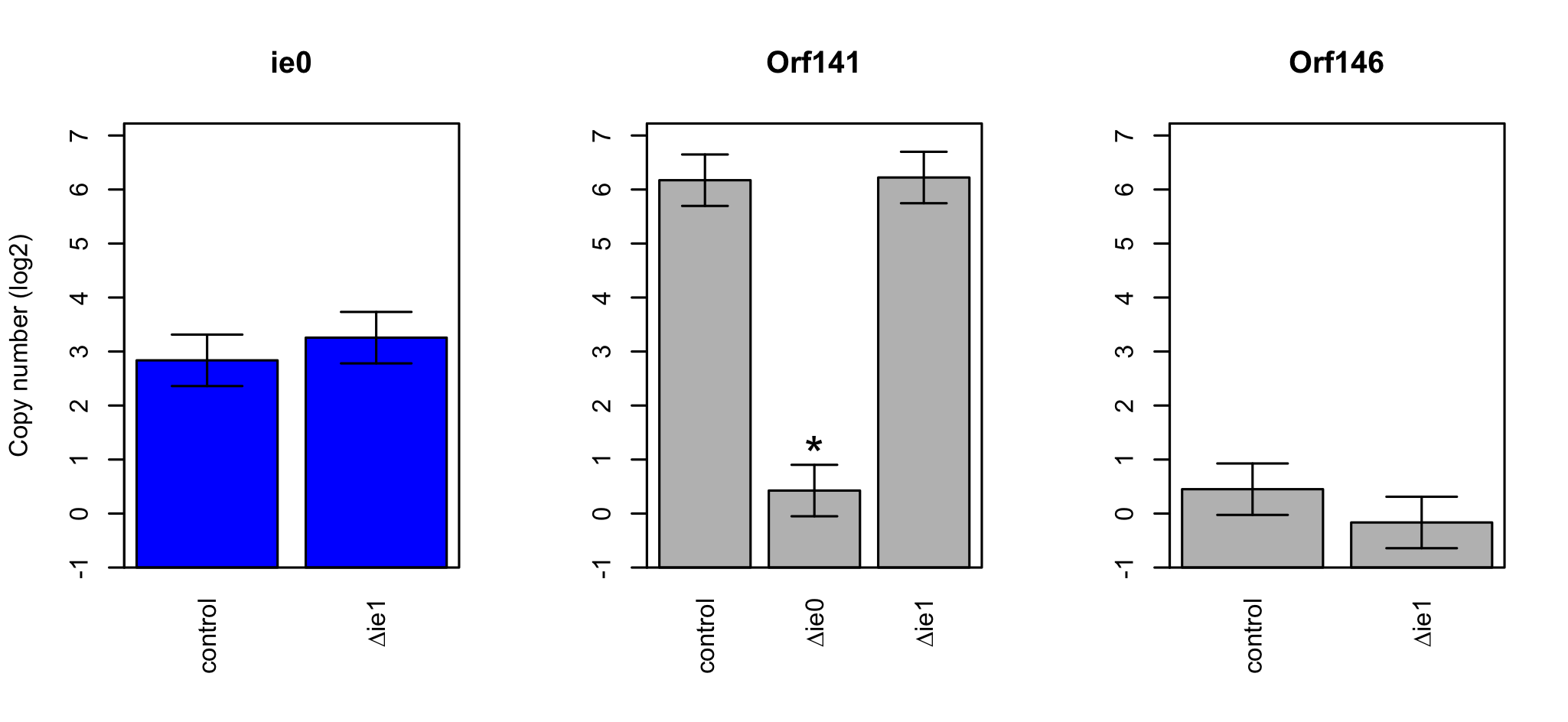

Supplement: S4 Fig — The abundance of mRNA of Orf141 or Orf146 in cells transfected with control, ie0, or ie1 gene knockout virus 6 hours post-transfection was quantified using real-time PCR. The estimated mean copy numbers of ie0, Orf141, or Orf146 transcript in cells transfected with bacmids indicated by the columns below the panel are shown. Expression of ie0 transcript was measured as a positive control for that of the late genes. The values are the sums of the estimated steady-state expression level in the control virus and the estimated genotype effect of the knockout virus. Error bars are standard errors of the estimated copy numbers. These values were estimated by fitting a mixed linear model. The asterisk (*) indicates significant regulatory functions: p-value < 0.05 in comparison to the control. The reported values are calculated from three technical replicates and three biological replicates. (TIF) [file pone.0119580.s004.tif]
