# Supplementary material for: Tightly Regulated Expression of Autographa californica Multicapsid Nucleopolyhedrovirus Immediate Early Genes Emerges from Their Interactions and Possible Collective Behaviors
Source: PLoS One. 2015 Mar 27;10(3):e0119580. doi: 10.1371/journal.pone.0119580 (PMC4376880; doi:10.1371/journal.pone.0119580)
Supplement: S1 Table — Restriction enzyme sites are underlined. (DOCX) [file pone.0119580.s005.docx]

| Table S1. List of nucleotide sequences for primers used in this study. Restriction enzyme sites are underlined. | | |  |
| --- | --- | --- | --- |
| **Purpose/target** | **Primer name** | **Primer sequence** | **Note** |
| **Construction of reporter plasmids** |  |  |  |
| *ie0* promoter and 5' UTR | Acie0p 5´ Bgl | AGATCTTTTTACTTACAATGCTGCT | BglII |
| *ie0* promoter and 5' UTR | Acie0p 3´ Hind | AAGCTTGTTGCGTTGCCCGTTATCA | HindIII |
| *ie1* promoter and 5' UTR | Acie1p 5´ Bgl | GTCGACGAACCGAAGAGAGAGCACT | BglII |
| *ie1* promoter and 5' UTR | Acie1p 3´ Hind | AAGCTTAGTCACTTGGTTGTTCACG | HindIII |
| *ie2* promoter and 5' UTR | Acie2p 5´ Bgl | AGATCTCAAGCGTAGGACGTTCATA | BglII |
| *ie2* promoter and 5' UTR | Acie2p 3´ Hind | AAGCTTGGCTGGGCTGGTAGGATAC | HindIII |
| *me53* promoter and 5' UTR | Acme53p 5´ Bgl | AGATCTTATCGTCGATCATTGCAAA | BglII |
| *me53* promoter and 5' UTR | Acme53p 3´ Hind | AAGCTTTGTAACTGTTAGTTAGCAC | HindIII |
| *pe38* promoter and 5' UTR | Acpe38p 5' Bgl | AGATCTGAGACAGCCTGTGGCGGCG | BglII |
| *pe38* promoter and 5' UTR | Acpe38-upR Hind | AAGCTTTTTGCTTATTGGCAGGCTC | HindIII |
| **Construction of IE expression plasmids.** |  |  |  |
| *ie1* expression cassette | Acie1p 5´ Bgl | AGATCTGTCGGGTCCATTGTCC | BglII |
| *ie1* expression cassette | Acie1 3' Pst | CTGCAGTTAATTAAATTCGAATTTT | PstI |
| *ie2* expression cassette | Acie2p 5´ Bgl | AGATCTCAAGCGTAGGACGTTCATA | BglII |
| *ie2* expression cassette | Acie2 3' Pst | CTGCAGTTAACGTCTAGACATAACA | PstI |
| *me53* expression cassette | Acme53p 5´ Bgl | AGATCTTATCGTCGATCATTGCAAA | BglII |
| *me53* expression cassette | Acme53 3' Pst | CTGCAGTTAGACATTGTTATTTACA | PstI |
| *pe38* expression cassette | Acpe38p 5' Bgl | AGATCTGAGACAGCCTGTGGCGGCG | BglII |
| *pe38* expression cassette | Acpe38 3' Pst | CTGCAGTTAATTTTCAAACCCAAAA | PstI |
| *ie0* expression cassette | Acie0 5' Hind | AAGCTTATGATAAGAACCAGCAGTC | BglII |
| *ie0* expression cassette | Acie1 3' Pst | CTGCAGTTAATTAAATTCGAATTTT | HindIII |
| **Suppression of IE1 translation** |  |  |  |
|  | Acie0 5' Hind | AAGCTTATGATAAGAACCAGCAGTC | BglII |
|  | Acie1 3' Pst | CTGCAGTTAATTAAATTCGAATTTT | HindIII |
|  | Acie0-M55A | CCAAGTGACTGCGACGCAAAT | The mutation from initiation codon to alanine is undelined. |
| **Construction of IE KO bacmids** |  |  |  |
| *ie0* promoter and 5' UTR | Acie0p 5´ Bgl | AGATCTTTTTACTTACAATGCTGCT | BglII |
| *ie0* promoter and 5' UTR | Acie0p 3´ Hind | AAGCTTGTTGCGTTGCCCGTTATCA | HindIII |
| *ie0* 3' UTR | Acie0-downF Sal | GTCGACGTAATGATCGATAACTTTG | SalI |
| *ie0* 3' UTR | orf142 300 | TTCTAAAATGTCCACAGTTG |  |
| *ie1* promoter and 5' UTR | Acie1p 5´ Bgl | GTCGACGAACCGAAGAGAGAGCACT | BglII |
| *ie1* promoter and 5' UTR | Acie1p 3´ Hind | AAGCTTAGTCACTTGGTTGTTCACG | HindIII |
| *ie1* 3' UTR | Acie1-downF Sal | GTCGACGAACCGAAGAGAGAGCACT | SalI |
| *ie1* 3' UTR | Acie1-downR | CTTCCTGCGGGCCAAACACT |  |
| *ie2* promoter and 5' UTR | Acie2p 5´ Bgl | AGATCTCAAGCGTAGGACGTTCATA | BglII |
| *ie2* promoter and 5' UTR | Acie2p 3´ Hind | AAGCTTGGCTGGGCTGGTAGGATAC | HindIII |
| *ie2* 3' UTR | Acie2-downR Sal | GTCGACAGAGTTTGAGCGCAGTAAC | SalI |
| *ie2* 3' UTR | orf150 5´ | ATGTTAAAACCCAACAT |  |
| *me53* promoter and 5' UTR | Acme53p 5´ Bgl | AGATCTTATCGTCGATCATTGCAAA | BglII |
| *me53* promoter and 5' UTR | Acme53p 3´ Hind | AAGCTTTGTAACTGTTAGTTAGCAC | HindIII |
| *me53* 3' UTR | Acme53-downR Sal | GTCGACTCATGTATTACTTTGAAGC | SalI |
| *me53* 3' UTR | orf138 300 | GTCTCTGAGGTCTGCG |  |
| *pe38* promoter and 5' UTR | Acpe38p 5' Bgl | AGATCTGAGACAGCCTGTGGCGGCG | BglII |
| *pe38* promoter and 5' UTR | Acpe38-upR Hind | AAGCTTTTTGCTTATTGGCAGGCTC | HindIII |
| *pe38* 3' UTR | Acpe38-downF Sal | GTCGACGCTGCTAGTTCATGTGATG | SalI |
| *pe38* 3' UTR | orf154 300 | TGTAAAACACAATCGAGGGA |  |
| **Resue of polyhedrin gene or polyhedrin-*ie0M55A construct*** | |  |  |
|  | polh-F | CTCGAGGCGTAGAAGGAAAAAATAATG | XhoI |
|  | polh-R | CTCGAGGGTTGGGACGACG | XhoI |
|  | ORF008-5 | ATGCCGATTATTCATA |  |
|  | ORF008-300 | GGTCCAAGTTTCACGCA |  |
|  | ORF141-5 | ATGATAAGAACCAGCAGT |  |
|  | ORF147-300 | GGATTCCGAATAATAAGC |  |
| **Quantification of IE gene mRNA** |  |  |  |
|  | ie0-qPCR F | ACGTCAAACTGTGCGTCATC |  |
|  | ie0-qPCR R | GGTTGTTCACGATCTTGTCG |  |
|  | ie1-qPCR F | GAGCATTCTGCTGGTGTGAA |  |
|  | ie1-qPCR R | GTTAAACTGGCCCACCACAC |  |
|  | ie2-qPCR F | GGCTCTGCAACGAGGTTTAG |  |
|  | ie2-qPCR R | CAAATCTGGCGACTGTGGTA |  |
|  | me53-qPCR F | AAATCACAAAGAGCCCAACG |  |
|  | me53-qPCR R | CAGTTGACGTCATCCACCAC |  |
|  | pe38-qPCR F | GCAAGACAATTTGGACAGCA |  |
|  | pe38-qPCR R | ATTTGTATGGAGCGGAGCTG |  |
|  | late-ie0-qPCR F | CTTGAAACAGTTGCGAGACG |  |
|  | late-ie0-qPCR R | CTCGTTCACGTACATTTGCAT |  |
| **Quantification of late gene mRNA** |  |  |  |
|  | Orf141_F3 | AAAGCCAAAAGAATGTTGCG |  |
|  | Orf141_R3 | TTATACGATGTCCTGCACGC |  |
|  | Orf-146_F0 | TCCGCCGACATACAATGTTA |  |
|  | Orf-146_R0 | AGCTGATCACGTACGCTCCT |  |
